# The association of neck circumference with incident congestive heart failure and coronary heart disease mortality in a community-based population with or without sleep-disordered breathing

**DOI:** 10.1186/s12872-018-0846-9

**Published:** 2018-05-31

**Authors:** Jingjing Zhang, Qi Guo, Liyuan Peng, Jiamei Li, Ya Gao, Bin Yan, Bangjiang Fang, Gang Wang

**Affiliations:** 1 Department of Emergency Medicine, the Second Affiliated Hospital of Xi’an Jiaotong University, Xi’an, 710004 China; 2 Department of Cardiology, the Second Affiliated Hospital of Xi’an Jiaotong University, Xi’an, China; 3 Division of Cardiology, Department of Internal Medicine, Tongji Hospital, Tongji Medical College, Huazhong University of Science & Technology, Wuhan, China; 4 Clinical Research Centre, the First Affiliated Hospital of Xi’an Jiaotong University, Xi’an, China; 5 Department of Emergency Medicine, Longhua Hospital, Shanghai University of Traditional Chinese Medicine, Shanghai, China

**Keywords:** Neck circumference, Congestive heart failure, Coronary heart disease, Mortality, Sleep-disordered breathing

## Abstract

**Background:**

Neck circumference (NC), representing upper body subcutaneous adipose tissue, may be correlated with increased risk of overweight/obesity, obstructive sleep apnoea, and metabolic and cardiovascular disease. However, the relationship between NC and the incidence of congestive heart failure (CHF) or mortality due to coronary heart disease (CHD) in a community-based population with and without sleep-disordered breathing (SDB) has not yet been clarified.

**Methods:**

We performed a prospective study using the Sleep Heart Health Study (SHHS) cohort. Cox proportional hazards regression models were used to estimate the association of different levels of NC with CHF incidence or CHD mortality in 2234 individuals with SDB and 2199 without SDB, respectively.

**Results:**

After adjusting for age, sex, and body mass index (BMI), NC was significantly associated with CHF when comparing the highest NC quartile group with the lowest (hazard ratio, HR, 2.265, 95% confidence interval, CI, 1.074–4.777) in the non-SDB population. This association diminished after further adjustment for other risk factors, but remained statistically significant, with an adjusted HR of 1.082 (95% CI 1.003–1.166) per unit increase in NC. Additionally, after adjustment for age, sex, and BMI, NC was also shown to be remarkably associated with CHD mortality (HR 1.141, 95% CI 1.014–1.282) per unit increase in NC in the non-SDB population but not in the SDB population. After adjustment for all the covariates, there was a significant association between NC and CHD death in those without SDB, with an adjusted HR of 1.134 (95% CI 1.001–1.284) per unit increase in NC.

**Conclusions:**

NC may correlate with CHF incidence and CHD mortality in population without SDB. NC measurement may help risk stratification for cardiovascular diseases.

**Trial registration:**

NCT00005275, January 1994.

**Electronic supplementary material:**

The online version of this article (10.1186/s12872-018-0846-9) contains supplementary material, which is available to authorized users.

## Background

Obesity, a pathological condition with body mass index (BMI) over 30 kg/m^2^, is associated with a series of metabolic risk factors [1]. However, the overall obesity includes unique intrinsic characteristics of different fat depots and the pattern of body fat distribution may also contribute to the prognosis [2]. Recently, significant heterogeneity was found between visceral adipose tissue (VAT) and subcutaneous adipose tissue (SAT) of the upper body. VAT is recognized as a pathogenic fat deposit with increased risk for insulin resistance, type II diabetes mellitus, and atherosclerosis [3, 4]. It was found that while lower body SAT may reduce the risk for cardiovascular disease, abdominal SAT was not related [5].

Previous studies have demonstrated that upper body fat produced the majority of systemic free fatty acids (FFA), which was associated with insulin resistance, increased very low-density lipoprotein, and endothelial dysfunction [3]. Neck circumference (NC), measured at the level of the laryngeal prominence, represents upper body SAT and may be correlated with increased risk of overweight/obesity, obstructive sleep apnoea, type II diabetes, metabolic syndrome, and cardiovascular disease [3, 4, 6–10]. However, the relationship between NC and the incidence of congestive heart failure (CHF) or mortality due to coronary heart disease (CHD) has not yet been clarified. Previously, the Sleep Heart Health Study (SHHS) was carried out in a geographically diverse, community-based population to assess the difference in cardiovascular outcomes among participants with different sleep apnoea statuses [11]. Based on SHHS, the present study aimed to investigate the role of NC in the incidence of CHF or CHD mortality in populations with and without sleep-disordered breathing (SDB).

## Methods

### Study design

Analyses were carried out in two subsets of data obtained from the participants of SHHS (ClinicalTrials.gov Identifier: NCT00005275). A total of 5804 participants aged 40 years and older underwent home polysomnography and completed a set of questionnaires on general health and sleep habits since 1994 [12]. Cardiovascular disease outcomes were tracked until 2010. Detailed aims and design of SHHS are described elsewhere [11]. The protocol was approved by the Institutional Review Board of each participating institution and signed informed consents were provided by the subjects. The data were accessed based on a signed agreement with the Brigham and Women’s Hospital.

### Participants

A total of 5804 subjects from SHHS cohort included 1915 from the Atherosclerosis Risk in Communities study, 1230 from the Cardiovascular Health Study, 688 from Framingham Offspring Cohort, and 1971 from other studies. Of them, 600 subjects were excluded because of pre-existing CHD, heart failure, or other related cardiovascular events, 762 because of lack of follow-up data, and 9 because of lack of NC data, ultimately leaving an analytical sample size of 4433. SDB has been proposed as a risk factor for cardiovascular events; besides, SHHS mainly focused on the relationship between SDB and cardiovascular outcomes. Therefore, our investigation was carried out in the following two groups: the group with SDB and the group without SDB (Additional file 1: Figure S1).

### NC and SDB

NC was measured during health interviews at the beginning of the observation period. Participants were advised to sit upright and look straight ahead. An inelastic tape was applied around the neck just below the laryngeal prominence. The NC measurement was made perpendicular to the long axis of the neck with the tape contacting the skin surface under acceptable pressure. Subjects were divided into NC quartiles of the following ranges: ≤34.1 (reference), 34.1–37.0, 37.0–40.5, and > 40.5 cm [13]. Home polysomnography was performed with the Compumedics P-series portable monitor (Abbotsford, Victoria, Australia). Detailed information were collected as described in a previously published work [14]. Apnoea was defined as complete or almost complete cessation of airflow lasting for at least 10 s, while hypopnea was defined as a clearly discernible decrease in airflow, or chest or abdominal plethysmograph amplitude that lasted for at least 10 s. Both, apnoea and hypopnea, required an associated 4% or greater oxyhaemoglobin desaturation. AHI is the average number of episodes of apnoea and/or hypopnea per hour of sleep [15]. SDB was defined as AHI ≥ 5 respiratory events per hour while non-SDB was defined as AHI < 5.

### CHD death and CHF

Baseline CHD or CHF was defined to be present if the participant responded positively to a standardized questionnaire before the polysomnogram monitoring, or reported that they had undergone coronary bypass surgery or coronary angioplasty, or if the parent cohort had an identified CHD event or CHF before the SHHS baseline. CHD death was defined as fatal CHD at any time between the baseline polysomnogram and the final follow-up between 2008 and 2011. Incident CHF was defined as the first occurrence of heart failure during this period of follow-up. Ongoing surveillance for CHD deaths and incident CHF events was carried out by the parent cohorts according to cohort-specific protocols.

### Other covariates

Smoking status was classified as “never” (if the participant reported lifetime smoking of fewer than 20 packs of cigarettes), “former”, or “current”. Educational level was classified as “less than 10 years”, “11–15 years”, “16–20 years” or “more than 20 years”. Diabetes was considered present if the participant was taking insulin or an oral hypoglycaemic agent. Hypertension was defined as systolic blood pressure ≥ 140 or diastolic blood pressure ≥ 90 mmHg or current use of antihypertensive medications [16]. Other covariates obtained from the parent cohorts were age, sex, race, BMI, and levels of serum triglycerides and high-density lipoprotein cholesterol.

### Statistical analysis

Data is presented as mean ± standard deviation for continuous variables and number (percentage) for categorical variables. Cox proportional hazards models were used to evaluate the association between NC and incident CHF or CHD mortality. The overall significance of the association of NC with each outcome was tested with NC modelled as a continuous variable. Survival time was defined as the time from baseline polysomnogram to the first CHF (or CHD death) event. Censoring time was the time of last known disease-free status for those without cardiovascular disease. All analyses were adjusted in the model for the following covariates: (1) age, sex, and BMI; (2) age, sex, BMI, and waist circumference; (3) age, sex, BMI, waist circumference, AHI, smoking status, serum levels of total cholesterol, triglycerides and high-density lipoprotein, history of diabetes, and history of hypertension, which are common risk factors for cardiovascular diseases. The relationship between different quartile levels of NC and the incidence of CHF or CHD mortality is presented as hazard ratio (HR) [95% confidence intervals (CI)]. Net reclassification improvement (NRI) index and integrated discrimination improvement (IDI) index were calculated to assess the incremental value of NC in predicting cardiovascular diseases. *P* values < 0.05 were considered to be statistically significant. Statistical analyses were performed with SPSS software (version 22.0, IBM Corp., Armonk, NY, USA) as well as with R (version 3.0.1, R Foundation for Statistical Computing, Vienna, Austria).

## Results

A total of 4433 subjects without CHD or CHF at baseline were followed up for a median of 10.9 years. It was a predominantly Caucasian population (86.6%) with an average age of 63 years (results not displayed). The group without SDB was younger (60.93 ± 11.05 years) and had more female patients (67.4%), lower BMI, and less cardiovascular risks such as dyslipidaemia, abnormal blood pressure, and diabetic status than the group with SDB (*P* < 0.05) (Additional file 2: Table S1). More baseline characteristics are shown in Additional file 3: Table S2.

**Fig. 1:**
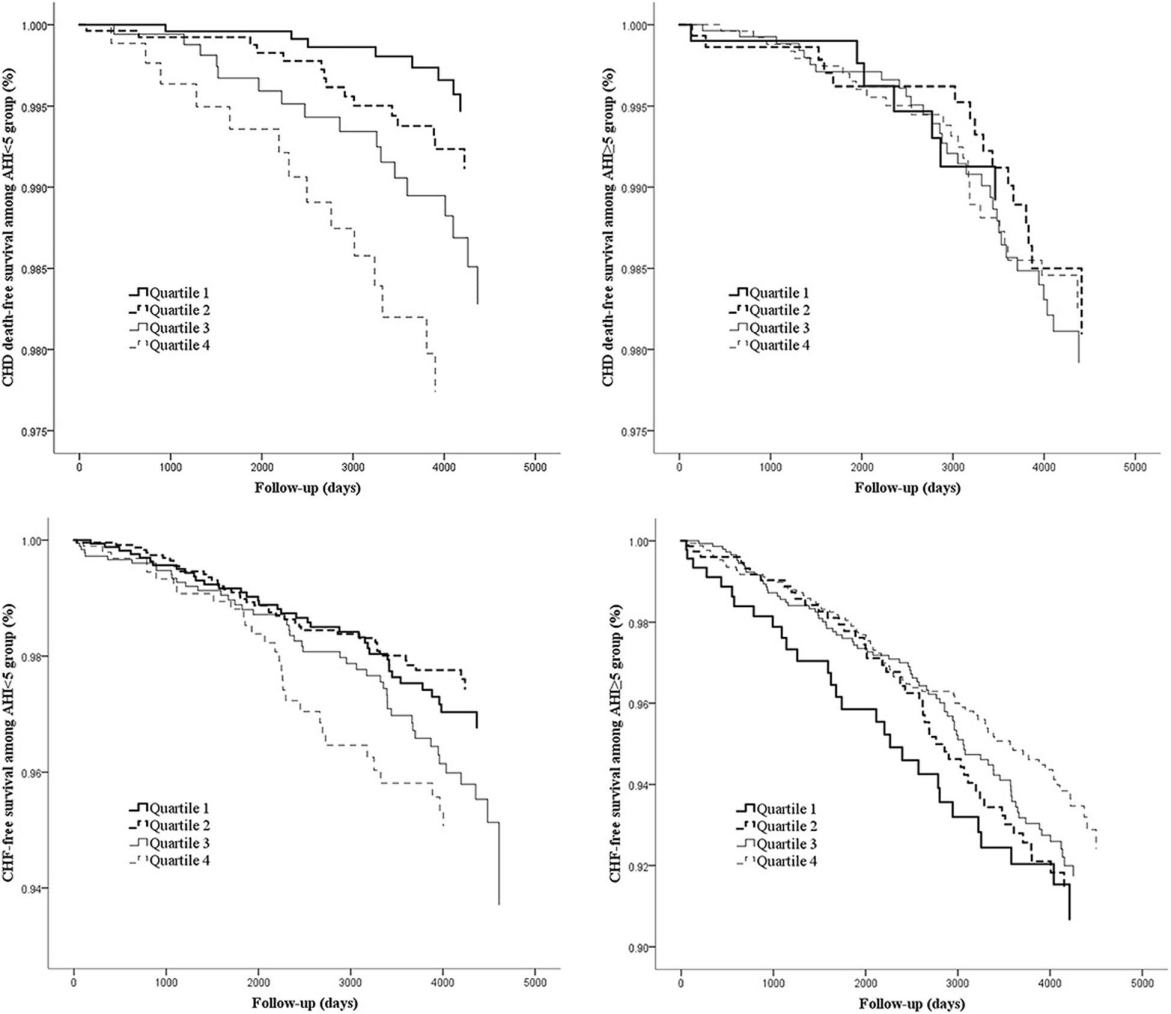
Adjusted Kaplan-Meier survival curves for NC quartiles, according to the AHI group and event type. All the results were adjusted for age, sex, BMI, waist circumference, AHI, smoking status, total cholesterol, triglycerides, high-density lipoprotein, history of diabetes, and history of hypertension. CHF = congestive heart failure, CHD = coronary heart disease, AHI = apnoea-hypopnea index, BMI = body mass index, NC = neck circumference

During the follow-up period, 163 CHF events and 58 CHD deaths were observed in the group with non-SDB, and 251 and 86 in the group with SDB, respectively. There was a positive linear association between NC quartiles and CHF incidence in the group without SDB (*P* = 0.027), while no association was observed in the SDB group. The same relationship also existed between NC and CHD mortality in the group without SDB (*P* = 0.003) (Additional file 4: Figure S2). Survival analysis also revealed the difference in the outcomes between these two subgroups. It was found that the CHD death-free curve in the group without SDB had greater separation and more obvious consistency with NC quartiles than the one in the SDB group. In addition, the tendency for a higher incidence of CHF with increasing NC quartiles existed in the group without SDB only (Fig. 1).

In the group without SDB, after adjustment for age, sex, and BMI, the HR for the incidence of CHF was 2.265 (95% CI 1.074–4.777) in the highest NC quartile compared with that in the lowest NC quartile. After further adjustment for waist circumference and other variables, the association was diminished and became not significant. However, the association of NC, as a continuous variable, with CHF incidence remained significant with an adjusted HR of 1.082 (95%CI 1.003–1.166) per unit increase in NC after even adjusting for all of the variables in the group without SDB (*P* = 0.040). In the SDB group, the association was not statistically significant in both the univariate and multivariate models (Table 1).

**Table 1:** The association of incident CHF with neck circumference according to AHI categories

	Quartiles of neck circumference				
	Q1 (low)	Q2	Q3	Q4 (high)	Overall tendency
AHI < 5^#^					
Subjects, n	805	655	491	283	
Events, n (%)	39 (4.8)	45 (6.9)	49 (10.0)	30 (10.6)	
Univariate Model	1 (Ref)	1.457 (0.949–2.238)	2.197 (1.443–3.346)	2.341 (1.455–3.769)	1.095 (1.054–1.137)
Multivariate Model 1	1 (Ref)	0.997 (0.630–1.577)	1.738 (0.982–3.077)	2.265 (1.074–4.777)	1.111 (1.034–1.194)
Multivariate Model 2	1 (Ref)	0.891 (0.561–1.415)	1.386 (0.780–2.463)	1.762 (0.850–3.650)	1.080 (1.008–1.157)
Multivariate Model 3	1 (Ref)	0.844 (0.522–1.364)	1.419 (0.778–2.589)	1.668 (0.765–3.638)	1.082 (1.003–1.166)
AHI ≥ 5^*^					
Subjects, n	304	455	681	759	
Events, n (%)	31(10.2)	51(11.2)	83(12.2)	86(11.3)	
Univariate Model	1 (Ref)	1.086 (0.695–1.697)	1.196 (0.792–1.807)	1.107 (0.734–1.669)	1.004 (0.974–1.035)
Multivariate Model 1	1 (Ref)	1.051 (0.662–1.670)	1.065 (0.646–1.757)	1.107 (0.600–2.041)	0.999 (0.948–1.054)
Multivariate Model 2	1 (Ref)	0.972 (0.609–1.550)	0.969 (0.587–1.599)	0.995 (0.544–1.820)	0.996 (0.947–1.049)
Multivariate Model 3	1 (Ref)	0.870 (0.533–1.418)	0.807 (0.480–1.357)	0.690 (0.364–1.309)	0.965 (0.912–1.022)

Model 1 adjusted for age, gender, and BMI

Model 2 adjusted for age, gender, BMI, and waist circumference

Model 3 adjusted for age, gender, BMI, waist circumference, AHI, smoking status, total cholesterol, triglycerides, high-density lipoprotein, history of diabetes, and history of hypertension

Results are presented as hazard ratio (95% confidence interval). *CHF* congestive heart failure, *AHI* apnoea-hypopnea index (the average number of episodes of apnoea and/or hypopnea per hour of sleep), *BMI* body mass index

^#^*P* value for the overall tendency of neck circumference modelled as a continuous variable was <0.001, 0.004, 0.029, 0.040 in the univariate model, multivariate model 1, model 2, and model 3, respectively

*P value for the overall tendency of neck circumference modelled as a continuous variable was 0.809, 0.978, 0.893, and 0.225 in univariate model, multivariate model 1, model 2, and model 3, respectively

Unlike the relationship between CHF and NC quartile, the HR for the incidence of fatal CHD was 5.477 (95% CI 1.422-21.090) in the highest NC quartile compared with that in the lowest NC quartile after adjusting for all of the variables in the group without SDB. In addition, after adjustment for age, sex, and BMI, the HR of NC, as a continuous variable, for CHD death was 1.141 (95% CI 1.014–1.282) and 1.061 (0.979–1.150) in the groups with and without SDB, respectively. In the model with additional adjustment for waist circumference, the association between fatal CHD and NC was significant only in the group without SDB (*P* = 0.022). With all the covariates adjusted in multivariate model 3, there was still a significant association between NC and CHD death in those without SDB, with an adjusted HR of 1.134 (95% CI 1.001–1.284) per unit increase in NC (Table 2).

**Table 2:** The association of CHD death with neck circumference according to AHI categories

	Quartiles of neck circumference				
	Q1 (low)	Q2	Q3	Q4 (high)	The overall tendency
AHI < 5^#^					
Subjects, n	805	655	491	283	
Events, n (%)	10 (1.2)	17 (2.6)	17 (3.5)	14 (4.9)	
Univariate Model	1 (Ref)	2.120 (0.971–4.631)	2.951 (1.351–6.445)	4.164 (1.849–9.374)	1.117 (1.049–1.189)
Multivariate Model 1	1 (Ref)	1.622 (0.710–3.705)	2.760 (0.977–7.798)	5.480 (1.574–19.075)	1.141 (1.014–1.282)
Multivariate Model 2	1 (Ref)	1.629 (0.714–3.717)	2.782 (0.992–7.807)	5.486 (1.624–18.527)	1.139 (1.019–1.274)
Multivariate Model 3	1 (Ref)	1.914 (0.762–4.807)	3.427 (1.806–10.814)	5.477 (1.422–21.090)	1.134 (1.001–1.284)
AHI ≥ 5^*^					
Subjects, n	304	455	681	759	
Events, n (%)	8 (2.6)	18 (4.0)	34 (5.0)	26 (3.4)	
Univariate Model	1 (Ref)	1.479 (0.643–3.402)	1.886 (0.873–4.074)	1.291 (0.584–2.851)	1.013 (0.962–1.068)
Multivariate Model 1	1 (Ref)	1.773 (0.749–4.194)	2.115 (0.858–5.215)	1.972 (0.663–5.868)	1.061 (0.979–1.150)
Multivariate Model 2	1 (Ref)	1.643 (0.690–3.914)	1.891 (0.762–4.690)	1.735 (0.586–5.135)	1.050 (0.968–1.139)
Multivariate Model 3	1 (Ref)	1.292 (0.504–3.309)	1.538 (0.579–4.087)	1.393 (0.472–4.538)	1.033 (0.944–1.130)

Model 1 adjusted for age, gender, and BMI

Model 2 adjusted for age, gender, BMI, and waist circumference

Model 3 adjusted for age, gender, BMI, waist circumference, AHI, smoking status, total cholesterol, triglycerides, high-density lipoprotein, history of diabetes, and history of hypertension

Results are presented as hazard ratio (95% confidence interval). *CHD* coronary heart disease, *AHI* apnoea-hypopnea index, *BMI* body mass index

^#^*P* value for the overall tendency of neck circumference modelled as a continuous variable was 0.001, 0.028, 0.022, and 0.048 in univariate model, multivariate model 1, model 2, and model 3, respectively

*P value for the overall tendency of neck circumference modelled as a continuous variable was 0.616, 0.149, 0.236, and 0.478 in univariate model, multivariate model 1, model 2, and model 3, respectively

To further evaluate whether NC has an incremental value in predicting the risk of CHF and CHD death, IDI and NRI were calculated in our study (Table 3). Reclassification statistics showed a significant improvement in NRI index of 0.2017 (*P* = 0.0135), indicating that, with the addition of NC, the prediction power of the model adjusted for age, sex, BMI has been improved, and the proportion of correct classification increased by 20.17%. The prediction for CHD death were also improved (NRI = 0.2655, *P* = 0.0460). At the meantime, the IDI index was 0.0060 (*P* = 0.0447) in the model predicting CHD death in the non-SDB population, indicating that an aggregate measure of sensitivity and specificity was superior in the model adjusted for age, sex, BMI and NC compared with the one adjusted for age, sex and BMI only. However, both reclassification statistics showed no significant improvement in the predicting value with the addition of NC in the SDB population.

**Table 3:** Incremental value of neck circumference in predicting the risk of incident CHF and CHD death

		CHF	CHD death
AHI < 5	NRI	0.2017 (0.0420**–**0.3614)	0.2655 (0.0051–0.5260)
	*P*	0.0135	0.0460
	IDI	0.0055 (−0.0008–0.0118)	0.0060 (0.0001–0.0119)
	*P*	0.0885	0.0447
AHI ≥ 5	NRI	0.0081 (−0.1233–0.1395)	0.0862 (−0.1288–0.3012)
	*P*	0.9041	0.4331
	IDI	0.0003 (−0.0005–0.0012)	−0.0006 (− 0.0031–0.0019)
	*P*	0.3785	0.6398

Reclassification indices were calculated for the addition of neck circumference in the model adjusted for age, sex, and BMI

*AHI* apnoea-hypopnea index, *BMI* body mass index, *CHF* congestive heart failure, *CHD* coronary heart disease, *NRI* net reclassification improvement, *IDI* integrated discrimination improvement

## Discussion

The SHHS study was conducted in a community-based population and included ethnically diverse participants with AHI status. It has reported several valuable findings on the association between SDB and hypertension, CHD, and CHF [17–19]. To the best of our knowledge, our study is the first prospective cohort study to evaluate the association between NC and future CHF events and fatal CHD in a large cohort. This study indicated that participants without SDB with higher NC may develop more CHF events and CHD deaths. NC might be an early risk factor and showed preclinical predictive value for CHD death and CHF events in the population without SDB.

Body fat is mainly located beneath the skin, around the abdominal organs (stomach, liver, intestines, kidneys, etc.), within the bone marrow, and within muscles [20]. It has been reported that nearly all of the major cardiovascular risk factors, such as blood glucose level, plasma lipid ratio, and blood pressure worsen with obesity [21]. Conversely, low BMI has also been shown to be a risk factor for cardiovascular and bleeding events in a prospective observational study [22]. Therefore, BMI might be not capable of reflecting the characteristics of local fat deposition as different compartments of body fat have been demonstrated to be associated with heterogeneous physiological and pathological metabolism [23].

VAT accounts for 10–20% of the total body fat in men and 5–10% in women, and has been reported to predict cardiovascular diseases independent of traditional measures of obesity [24]. SAT, which is over 80% of the total body fat around the abdominal organs, buttocks, thighs, and neck, has drawn more attention in recent decades. These subcutaneous layers are functionally distinct and independently correlate with metabolic complications [25]. For example, gluteofemoral SAT was found to contribute to the long-term entrapment of excess fatty acids and protect from the adverse effects associated with ectopic fat deposition [26]. Additionally, several studies have reported that waist-to-hip ratio was strongly positively associated with cardiovascular outcomes in comparison with BMI [27, 28].

NC, as an anatomically separate component of the body, is a distinct fat depot that represents the upper body SAT. The Framingham Heart Study demonstrated that NC was a novel measurement for cardio-metabolic risk and associated with cardiovascular disease risk factors, including blood pressure, triglycerides, and fasting blood glucose [3]. However, there was no statistically significant association between NC and incidence of cardiovascular disease in the entire population according to their secondary analysis, while another study found that NC independently contributed to the prediction of cardio-metabolic risks that may be superior to waist circumference or BMI [29]. Our study has also shown that a higher NC may contribute to CHF as well as CHD mortality, but only in the non-SDB population, after adjusting for BMI and other cardiovascular risk factors.

The potential mechanism may involve elevated levels of plasma FFAs [30]. The FFAs in circulation could inhibit oxidation and glucose uptake, and impair insulin signalling transduction through a variety of pathways, leading to insulin resistance. In addition, FFAs are also associated with lipid metabolism disorder, vascular endothelial injury, and hypertension [31]. Upper body SAT, typically represented by NC, has been demonstrated to be associated with a much larger proportion of systemic FFAs [32]. Therefore, NC might be a much earlier signal of predicting cardiovascular disease (such as CHF or CHD death) amongst the anthropometric indicators. Additionally, as an index of upper body obesity, NC is always recognized as a simple and time saving measurement. Compared with waist circumference, NC is less affected by the changes in the body size caused by breathing, exercises, diet, and lifestyle habits such as drinking alcohol [33, 34]. The measurement of NC might be especially useful in special populations such as morbidly obese people, patients on bed rest, and pregnant women [34].

It should be noted that the predictive value of NC has not been found in individuals with AHI ≥ 5 in our study, although the baseline characteristics showed that the members of this group were older and suffered from higher BMI, NC, waist circumference, and other cardiovascular risks. It is proposed that the predictive value of NC level in the SDB group may be diminished by the strong association of SDB with NC and cardiovascular diseases. First, studies found that NC was significantly higher in patients with severe SDB than in those with non-severe SDB whether obese or not, possibly due to the increased mass of upper respiratory tract soft tissues [35, 36]. In addition, SDB and NC were demonstrated to be associated with lower levels of high-density lipoprotein and incidence of diabetes, which may directly contribute to cardiovascular diseases [34, 37, 38]. SDB may also cause cardiovascular diseases by sympathetic nervous system activation and systemic inflammation resulting from intermittent hypoxemia [39]. Therefore, a larger cohort of SDB and non-SDB population may be needed to further investigate the interactions between multiple risk factors.

This study has certain limitations that deserve discussion. Firstly, although NC is a simple and feasible anthropometric measurement, there are other factors that influence NC other than fat deposition. Further imaging studies on quantification of SAT of the neck are required to validate our study findings. Secondly, the small number of CHD death events in the people without SDB did not exceed the number of covariates by at least ten-times, which made the multivariate model 3 less powerful. Thirdly, as in an observational analysis, residual confounding by unmeasured variables, such as diet or physical activity, cannot be excluded, despite multivariate adjustment for existing risk factors.

## Conclusions

A higher NC might predict the incidence of CHF and CHD deaths in a community-based population without SDB. However, precise examination of the relationship between neck SAT on cardiovascular outcomes and the possible underlying mechanism is worth investigations in the future.

## Additional files

Additional file 1: **Figure S1.** Flow chart of the study sample. AHI = apnoea-hypopnea index. (TIF 1700 kb)

Additional file 2: **Table S1.** Characteristics of subjects between the population with SDB and non-SDB. (DOC 18 kb)

Additional file 3: **Table S2.** Characteristics of subjects by neck circumference quartiles. (DOC 59 kb)

Additional file 4: **Figure S2.** The relationship between NC and CHF or CHD death. There was a positive linear association between NC quartiles and CHF incidence or CHD mortality in the group without SDB, while no association was observed in the SDB group. The rate of outcome events was represented within each NC quartile according to AHI categories. AHI = apnoea-hypopnea index, CHF = congestive heart failure, CHD = coronary heart disease, NC = neck circumference. (TIF 2144 kb)

## Abbreviations

AHI: Apnoea-hypopnea index
BMI: Body-mass index
CHD: Coronary heart disease
CHF: Congestive heart failure
CI: Confidence interval
HR: Hazard ratio
IDI: Integrated discrimination improvement
NC: Neck circumference
NRI: Net reclassification improvement
SAT: Subcutaneous adipose tissue
SDB: Sleep-disordered breathing
SHHS: Sleep Heart Health Study
VAT: Visceral adipose tissue
