# Supplementary figures and images for: The association of neck circumference with incident congestive heart failure and coronary heart disease mortality in a community-based population with or without sleep-disordered breathing
Source: BMC Cardiovasc Disord. 2018 May 31;18:108. doi: 10.1186/s12872-018-0846-9 (PMC5984387; doi:10.1186/s12872-018-0846-9)

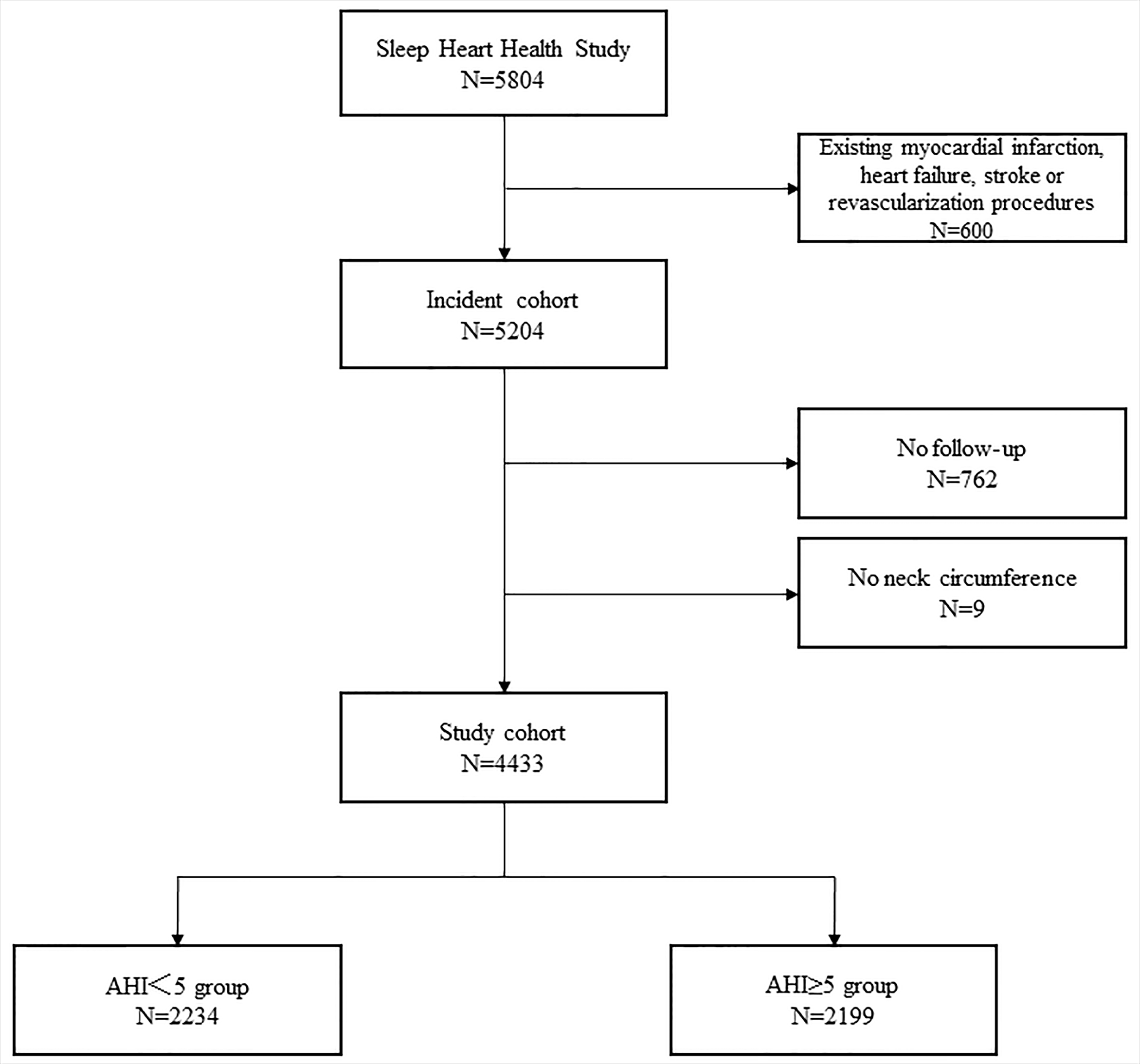

Supplement: Supplementary file 1 — Figure S1. Flow chart of the study sample. AHI = apnoea-hypopnea index. (TIF 1700 kb) [file 12872_2018_846_MOESM1_ESM.tif]

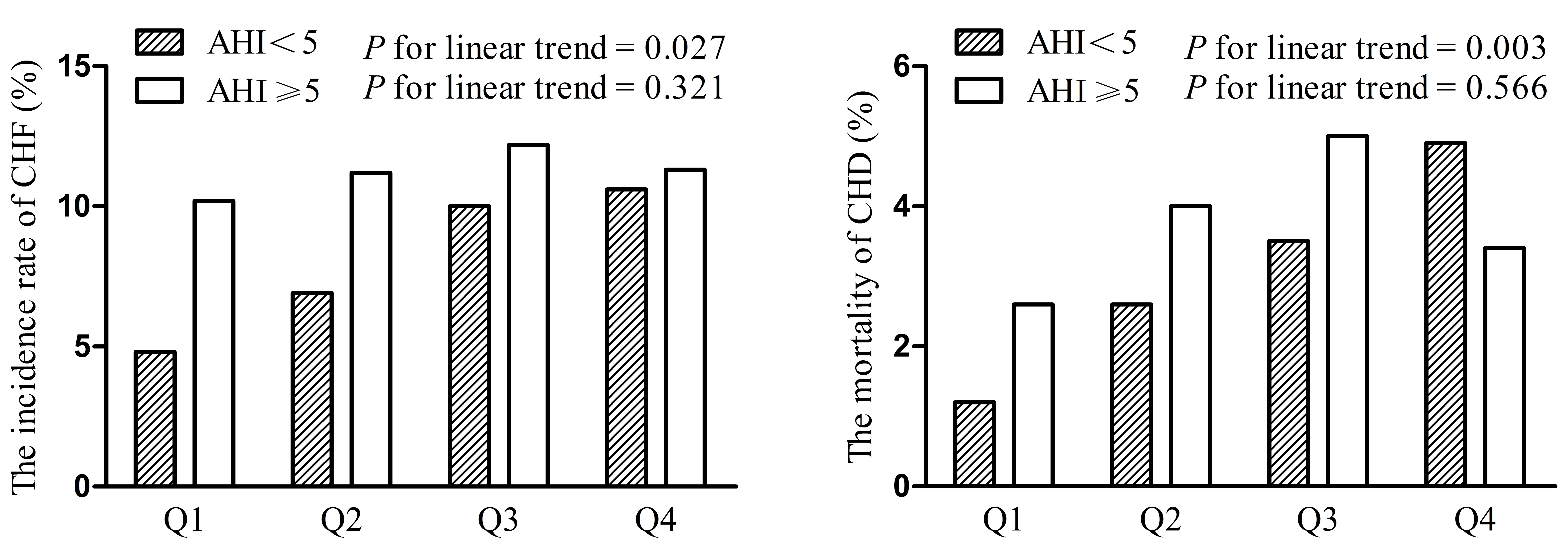

Supplement: Supplementary file 4 — Figure S2. The relationship between NC and CHF or CHD death. There was a positive linear association between NC quartiles and CHF incidence or CHD mortality in the group without SDB, while no association was observed in the SDB group. The rate of outcome events was represented within each NC quartile according to AHI categories. AHI = apnoea-hypopnea index, CHF = congestive heart failure, CHD = coronary heart disease, NC = neck circumference. (TIF 2144 kb) [file 12872_2018_846_MOESM4_ESM.tif]
