# Supplementary material for: The association of neck circumference with incident congestive heart failure and coronary heart disease mortality in a community-based population with or without sleep-disordered breathing
Source: BMC Cardiovasc Disord. 2018 May 31;18:108. doi: 10.1186/s12872-018-0846-9 (PMC5984387; doi:10.1186/s12872-018-0846-9)
Supplement: Supplementary file 2 — Table S1. Characteristics of subjects between the population with SDB and non-SDB. (DOC 18 kb) [file 12872_2018_846_MOESM2_ESM.doc]

| **Table S1** Characteristics of subjects between the population with SDB and non-SDB | | | |
| --- | --- | --- | --- |
|  | non-SDB group | SDB group | *P* |
| Subjects, n | 2234 | 2199 |  |
| Age, years | 60.93±11.05 | 65.19±10.51 | ＜0.001 |
| Male, n (%) | 729(32.6) | 1232(56.0) | ＜0.001 |
| Neck circumference, cm | 36.11±3.75 | 38.98±4.04 | ＜0.001 |
| Waist circumference, cm | 92.91±12.75 | 101.47±13.22 | ＜0.001 |
| BMI, kg/m2 | 26.90±4.40 | 29.70±5.28 | ＜0.001 |
| AHI, events/hour | 1.95±1.41 | 17.46±14.70 | ＜0.001 |
| Smoking status, n (%) |  |  | ＜0.001 |
| Never smoker | 1107(49.6) | 995(45.2) |  |
| Former smoker | 280(12.5) | 159(7.2) |  |
| Current smoker | 847(37.9) | 1045(47.5) |  |
| Total cholesterol, mg/dL | 206.13±38.77 | 207.75±37.66 | 0.169 |
| High-density lipoprotein, mg/dL | 53.66±16.48 | 48.83±14.76 | ＜0.001 |
| Triglycerides, mg/dL | 140.56±80.90 | 159.00±114.56 | ＜0.001 |
| History of diabetes, n (%) | 95(4.3) | 178(8.1) | ＜0.001 |
| History of hypertension, n (%) | 670(30.0) | 916(41.7) | ＜0.001 |
| CHF, n (%) | 163(7.3) | 251(11.4) | ＜0.001 |
| CHD death, n (%) | 58(2.6) | 86(3.9) | 0.014 |
| Follow-up time, days | 4049.87±1004.02 | 3923.95±1069.58 | ＜0.001 |

Results are presented as mean±standard deviation or number(percentage). CHD = coronary heart disease, CHF = congestive heart failure, AHI = apnoea-hypopnea index, BMI = body mass index.
