# Supplementary material for: The association of neck circumference with incident congestive heart failure and coronary heart disease mortality in a community-based population with or without sleep-disordered breathing
Source: BMC Cardiovasc Disord. 2018 May 31;18:108. doi: 10.1186/s12872-018-0846-9 (PMC5984387; doi:10.1186/s12872-018-0846-9)
Supplement: Supplementary file 3 — Table S2. Characteristics of subjects by neck circumference quartiles. (DOC 59 kb) [file 12872_2018_846_MOESM3_ESM.doc]

| **Table S2 Characteristics of subjects by neck circumference quartiles** | | | | | |
| --- | --- | --- | --- | --- | --- |
|  | **Quartiles of neck circumference** | | | |  |
|  | **Q1 (low)** | **Q2** | **Q3** | **Q4 (high)** | ***P*^#^** |
| Subjects, n | 1109 | 1110 | 1172 | 1042 |  |
| Age, years | 61.72±11.49 | 64.07±11.08 | 63.59±11.27 | 62.76±9.86 | ＜0.001 |
| Male, n (%) | 31 | 203 | 794 | 933 | ＜0.001 |
| Neck circumference, cm | 32.46±1.41 | 35.80±0.87 | 38.93±0.95 | 43.18±2.19 | ＜0.001 |
| Waist circumference, cm | 85.44±11.00 | 95.63±11.86 | 99.94±10.88 | 108.05±10.39 | ＜0.001 |
| BMI, kg/m2 | 24.66±3.48 | 27.97±4.38 | 28.79±4.67 | 31.92±4.78 | ＜0.001 |
| AHI, events/hour | 4.82±7.51 | 7.10±9.58 | 10.53±12.20 | 16.47±17.63 | ＜0.001 |
| Smoking status, n (%) |  |  |  |  | ＜0.001 |
| Never smoker | 641(57.79) | 599(53.96) | 512(43.68) | 350(33.58) |  |
| Former smoker | 350(31.55) | 407(36.66) | 546(46.58) | 589(56.52) |  |
| Current smoker | 118(10.64) | 104(9.36) | 114(9.72) | 103(9.88) |  |
| Race, n (%) |  |  |  |  | 0.063 |
| White | 958(86.38) | 950(85.58) | 1004(85.66) | 927(88.96) |  |
| Black | 62(5.59) | 84(7.56) | 81(6.91) | 60(5.75) |  |
| Other | 89(8.02) | 76(6.84) | 87(7.42) | 55(5.27) |  |
| Education level, n (%) |  |  |  |  | 0.020 |
| ≤10 years | 195(17.58) | 194(17.47) | 201(17.15) | 174(16.69) |  |
| 11-15 years | 551(49.68) | 569(51.26) | 577(49.23) | 495(47.50) |  |
| 16-20 years | 333(30.02) | 308(27.74) | 342(29.18) | 320(30.71) |  |
| ＞20 years | 30(2.70) | 39(3.51) | 62(5.29) | 53(5.08) |  |
| Total cholesterol, mg/dL | 207.74±37.65 | 211.98±39.66 | 205.28±38.13 | 202.69±36.80 | ＜0.001 |
| High-density lipoprotein, mg/dL | 60.39±16.49 | 54.78±15.89 | 47.33±12.43 | 42.35±11.68 | ＜0.001 |
| Triglycerides, mg/dL | 127.35±77.19 | 150.71±105.76 | 148.51±86.48 | 173.73±119.86 | ＜0.001 |
| History of diabetes, n (%) | 32(2.88) | 52(4.68) | 80(6.82) | 109(10.46) | ＜0.001 |
| History of hypertension, n (%) | 304(27.41) | 408(36.75) | 434(37.03) | 440(42.22) | ＜0.001 |
| CHF, n (%) | 70(6.31) | 96(8.64) | 132(11.26) | 116(11.13) | ＜0.001 |
| CHD death, n (%) | 18(1.62) | 35(3.15) | 51(4.35) | 40(3.83) | 0.002 |
| Follow-up time, days | 4049.87±1004.02 | 4014.48±1019.53 | 3923.95±1069.58 | 3944.69±1095.11 | 0.014 |

^#^*P* value for the comparison among neck quartiles by chi-square test or one-way analysis of variance. Data was presented as mean±standard deviation or n (percent). AHI, apnoea-hypopnea index; BMI, body-mass index; CHF, congestive heart failure; CHD, coronary heart disease.
